# Mosquitoes Eating Mosquitoes: How *Toxorhynchites amboinensis*, *Psorophora ciliata*, and *Sabethes cyaneus* (Diptera: Culicidae) Capture Prey

**DOI:** 10.1093/aesa/saac017

**Published:** 2022-10-04

**Authors:** Robert G Hancock, Taylor Boyd, Shannon MacFadden, Aaron Sowders, W A Foster, L P Lounibos

**Affiliations:** Department of Biology, Metropolitan State University of Denver, Campus Box 53, P.O. Box 3352, Denver, CO 80217-3362, USA; Department of Biology, Metropolitan State University of Denver, Campus Box 53, P.O. Box 3352, Denver, CO 80217-3362, USA; Department of Biology, Metropolitan State University of Denver, Campus Box 53, P.O. Box 3352, Denver, CO 80217-3362, USA; Middlesborough Middle School, Middleborough, KY 40965, USA; Department of Entomology, The Ohio State University, Columbus, OH 43210, USA; Florida Medical Entomology Laboratory, University of Florida, Vero Beach, FL 32962, USA

**Keywords:** microcinematography, aquatic, predation, behavior, evolution

## Abstract

High-speed microcinematography was used to elucidate the details of prey capture by the larvae of three predatory mosquito species. The obligate predators *Toxorhynchites amboinensis* and *Psorophora ciliata* exhibited a high degree of convergence as both utilize three essential elements: 1) abdominally-generated hemostatic pressure to propel the head towards the prey; 2) lateral palatal brushes (LPB) opening and fanning into anterior-directed basket-like arrangements; 3) simultaneously with the LPB-basket formation, the wide opening of sharp-toothed mandibles. Thus, LPBs and mandibles are used for prey capture by both species. The facultative predator *Sabethes cyaneus* utilizes a vastly different prey-capture mechanism that involves ventro-lateral body arching and scooping of prey with axially pointed siphons into the grasp of open maxillae bearing elongate apical teeth. Prey consumption, which is typically incomplete in this species, then involves the action of teeth on the mandibles which cut into the held prey. Although prey consumption is incomplete, simple experiments show that *Sa. cyaneus* do gain nutritionally from consuming mosquito larvae and that they do discriminate heterospecific from conspecific larvae and actively approach heterospecific mosquito prey. These findings indicate that independent evolutionary pathways have produced diverse predatory behaviors and morphologies in aquatic environments where the immature stages of mosquitoes co-occur.

Across the world, within the diverse range of water bodies where aquatic dipteran larvae occur, predation has evolved many times. This is most pronounced in the Infraorder Culicimorpha where 5 of the 8 assigned families include aquatic predators: *Prosiulium* in Simuliidae (Al-Shaer et al. 2015), *Pentaneura* in Chironomidae (Naeem 1988), all species but one in the Chaoboridae (Schremmer 1950, Marshall 2012), and the known larvae of all Corethrellidae (Borkent 2008) and in the large and diverse family Culicidae, where predators are found in 13 genera from both subfamilies and 5 of the 12 tribes currently recognized by Harbach (2007).

Predatory mosquitoes vary in their dependency on predation. Facultative predators (including cannibals) also feed on detritus, microbes, and/or other organic material by filtering or browsing (Surtees 1959). All members of the tribe Toxorhynchitini (subfamily Culicinae) belong to the genus *Toxorhynchites* (formerly *Megarhinus*), which are exclusively predatory (Breland 1949, Steffan and Evenhuis 1981, Russo 1986). In the tribe Culicini (subfamily Culicinae), *Psorophora* of the subgenus *Psorophora* are strictly predatory (while other subgenera of *Psorophora* are filterers and/or browsers) and all members of the genus *Lutzia* are obligate predators (Wilkerson et al. 2021). In the tribe Aedini, members of the subgenera *Alanstonea* and *Mucidus* include species that are predatory on other mosquitoes: *Aedes* (*Ala.*) *treubi* (deMeijere, Diptera: Culicidae) (Mogi & Chan 1996) and several species of *Aedes* (*Mucidus*) (Knight 1947). In the tribe Sabethini (subfamily Culicinae), facultative predation is common and diverse: *Sabethes* (Arnett, 1949, Galindo 1958), *Trichoprosopon* (Arnett 1949, Lounibos 1983), *Runchyomyia* (Talaga et al. 2016), and the *Wyeomyia* subgenus *Dendromyia* (Motta and Lourenco-de-Oliveira 2000) in the New World; and *Armigeres* (Bates 1949, Tanaka et al. 1979), *Eretmapodites* (Haddow 1946, Lounibos 1980), *Topomyia* (Mogi & Sembel 1996), and *Tripteroides* (Van den Assem 1959) in the Old World. While surface film filtering is the most common feeding mode in the subfamily Anophelinae, several species of *Anopheles* are facultative cannibals (Balfour 1921, Hinman 1934, Reisen and Emory 1976, Nannini and Juliano 1998, Koenraadt and Takken 2003). Thus, predation, both obligate and facultative, is a common and diverse phenomenon in the Culicidae.

As species of *Toxorhynchites* have been reared and released for the biological control of the immature stages of mosquito vectors and pests in container habitats (Steffan and Evenhuis 1981, Focks 2007), it is often presumed that mosquitoes are numerically important prey in the natural diets of these predatory larvae. However, dissections of the midguts of wild-caught larvae of *Toxorhynchites rutilus* (Coquillett, Diptera: Culicidae) from water-holding tires and treeholes to identify prey items from exoskeletal remains, revealed that mosquito immatures accounted for only 5–6% of total prey items captured and consumed in south Florida (Campos and Lounibos 2000a). No comparable studies have been performed on the natural diets of larvae of the three predatory species examined herein, but the presence of immatures of other species of mosquitoes in aquatic habitats occupied by *Psorophora ciliata* (Fabricius, Diptera: Culicidae) (Curtis 1985) and *Sabethes cyaneus* (Fabricius, Diptera: Culicidae) (Galindo et al. 1958) favors the presumption that immature Culicidae compose some proportion of the natural diets of these two species. For purposes of the current behavioral research, feeding all three predatory species a common diet of mosquito larvae facilitated the interspecific comparisons of prey capture mechanisms.

The ecology of predation and cannibalism is dynamically linked to the diversity of habitats where mosquitoes occur. Many predatory species are found in cavity and vessel habitats including phytotelmata and other natural and man-made containers. At the population and community levels predation may serve to prevent or reduce the deleterious effects of overcrowding which can negatively affect growth rate, adult size, and survivorship (e.g., Church and Sherratt 1996, Polis 1981, Campos and Lounibos 2000b).

The morphological and behavioral adaptations for predation are diverse and reflect the degree of dependence on it. Although detailed prey capture mechanisms are unresolved and most accounts are based on simple container studies pairing suspected predators with nonpredatory larvae, predatory species exhibit structural enhancements for capturing and consuming other aquatic larvae. Obligate predators (*Toxorhynchites*, *Lutzia*, and some *Psorophora*) have large heavily toothed mandibles (Galindo et al. 1958, Harbach and Knight 1980) and robust lateral palatal brushes (LPBs) often with rigid elements composed of fused setae that are unlike the flexible and numerous LPB-setae used for filtering in nonpredatory species. Facultative predators exhibit more variability in the structures used for grasping and manipulating prey, but common to all are the relatively well-developed mandibular teeth compared to nonpredatory species (Bates 1949). In the culicine tribe Sabethini the maxillae of predatory species often exhibit elongate apical teeth (Talaga et al. 2016).

Because most prior studies of mosquito predation lacked the equipment and techniques to resolve details at high resolution and in slow-motion, none of the prey-capture mechanisms has been previously described in anatomical detail although some postcapture, and thus slower, prey holding, and manipulation mechanisms have been described (Linley 1990). With the specific goal of elucidating the details in three different predatory taxa, we utilized high-speed 16 mm microcinematography and high speed macrovideography to record the strikes with great detail and clarity to describe the mechanisms of prey capture in the obligate predators *Toxorhynchites (Toxorhynchites) amboinensis* (Doleschall, Diptera: Culicidae) and *Ps. ciliata* and the facultative predator *Sa. cyaneus*.

## Materials and Methods

### Mosquito Sources and Rearing

*Tx. amboinensis*, a native of Southeast Asia and Oceania was obtained from a laboratory colony maintained at The Ohio State University. The source of the colony was unknown. Adults were held in cages containing various bottles with cotton wicks: some contained 20% honey and others contained water. Early instars were obtained weekly from black-painted plastic oviposition cups that were always in the cages. Larvae were raised individually in cells of ice-cube trays with daily additions of smaller *Aedes aegypti* (Linnaeus, Diptera: Culicidae) (Rockefeller Strain) larvae for food. *Psorophora* spp. larvae were collected from shallow irrigation ditches in citrus groves in Indian River County, Florida. Early instars were raised in the laboratory in white plastic photographic print-developing trays with other flood-plain larvae obtained from the original collection site, which included *Psorophora columbiae* (Dyar & Knab, Diptera: Culicidae) and *Aedes vexans* (Meigen, Diptera: Culicidae). To prevent cannibalism as prey became less abundant, *Ae. aegypti* (Vero Beach strain) larvae from laboratory colonies were added as needed. *Sabethes cyaneus* were obtained from a colony originally established from adult and larval collections taken by R. Hancock and W. Foster in 1988 from Majé Island in Lago Bayano Panama, and maintained at The Ohio State University. Finely ground food consisting of a 1:1:1 mixture of Purina Rat Chow, brewer’s yeast, and lactalbumin or finely ground Tetramin flakes were sprinkled into larval rearing pans as needed.

### High Speed Filming of *Tx. amboinensis* and *Ps. ciliata*

A Mitchell Monitor 16 High-Speed Camera, Model HS-16-E4 was connected to the phototube of a Zeiss Stemi SV stereomicroscope. Eastman Kodak 16mm color negative filmstock 7279 (500 A.S.A.) was used to record *Ps. ciliata* and *Tx. amboinensis* strikes at 340 frames per second (fps) and 0.75× magnification. Exposed filmstock was developed and transferred to Sony Betacam SP video tape for analysis on a color monitor. Strikes were induced by placing last instar predatory larvae into well slides with water followed by the presentation of a live prey larva which was grasped by the anal papillae with no. 5 jeweler’s forceps and placed in front of the predatory larva.

### High Speed Video of *Sa. cyaneus*

Predatory strikes were filmed at 2,531 fps and 4,352 fps using a Chronos HD 2.1 (Krontech, Burnaby, BC, Canada) camera with a Kern 75 mm Macro Switar lens and extension tubes. Lateral views were recorded in small acrylic aquaria with dimensions 1 cm (h) × 1.5 cm (w) × 1 cm (d). Underneath views were filmed in circular arenas made by cutting the bottom off of a 50 ml beaker with a diamond saw and placing the resultant “ring” on the bottom of a 10 gallon clear-glass aquarium filled with water to a depth of 2 cm. For both viewing angles for filming, individual fourth instar *Sa*. *cyaneus* larvae were combined with 1–4 larvae of *Aedes albopictus* (Skuse, Diptera: Culicidae) or *Ae. aegypti* of instars 2–4.

## Results

### Sa. cyaneus

**Fig. 1.**
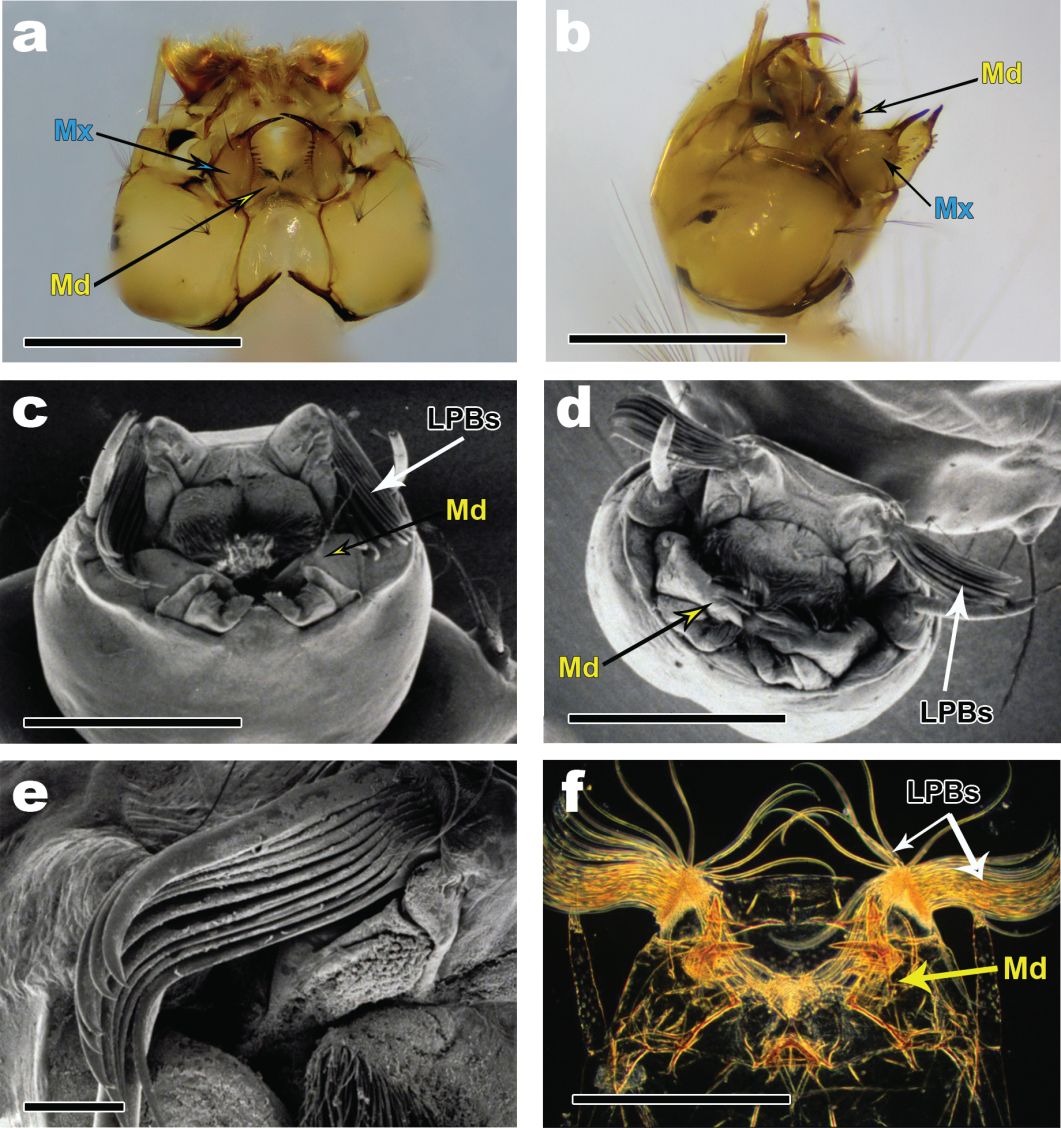
Head structures adapted for predation: (a) *Sa. cyaneus* – maxillae (Mx) with elongate apical teeth and toothed mandibles (Md) in closed positions; (b) *Sa. cyaneus* – maxillae (Mx) with elongate apical teeth and toothed mandibles (Md) in open (strike) positions; (c) *Tx. brevipalpis* – curved and toothed LPBs and heavily toothed mandibles (Md) in prestrike position; (d) *Tx. brevipalpis* – LPBs in open position before fanning into basket; (e) *Tx. brevipalpis* – detail of curved and unfanned LPBs; (f) *Ps. ciliata* – curved lateral palatal brushes (LPBs) and heavily-toothed mandibles (Md). (c–e) Used with permission from the Florida Entomological Society. Scale = 1 mm.

The principal structures involved in the capturing of prey include the mandibles and the maxillae on the head, the body trunk, and the siphon. Functional adaptations for predation include strongly-toothed mandibles for tearing, caliper-like and toothed maxillae for prey holding and manipulation, and elongate tapering siphons for snaring (Fig. 1a and b) (Supp Videos S1–5 [online only]).

### *Sa. cyaneus* Prestrike Position

**Fig. 2.**
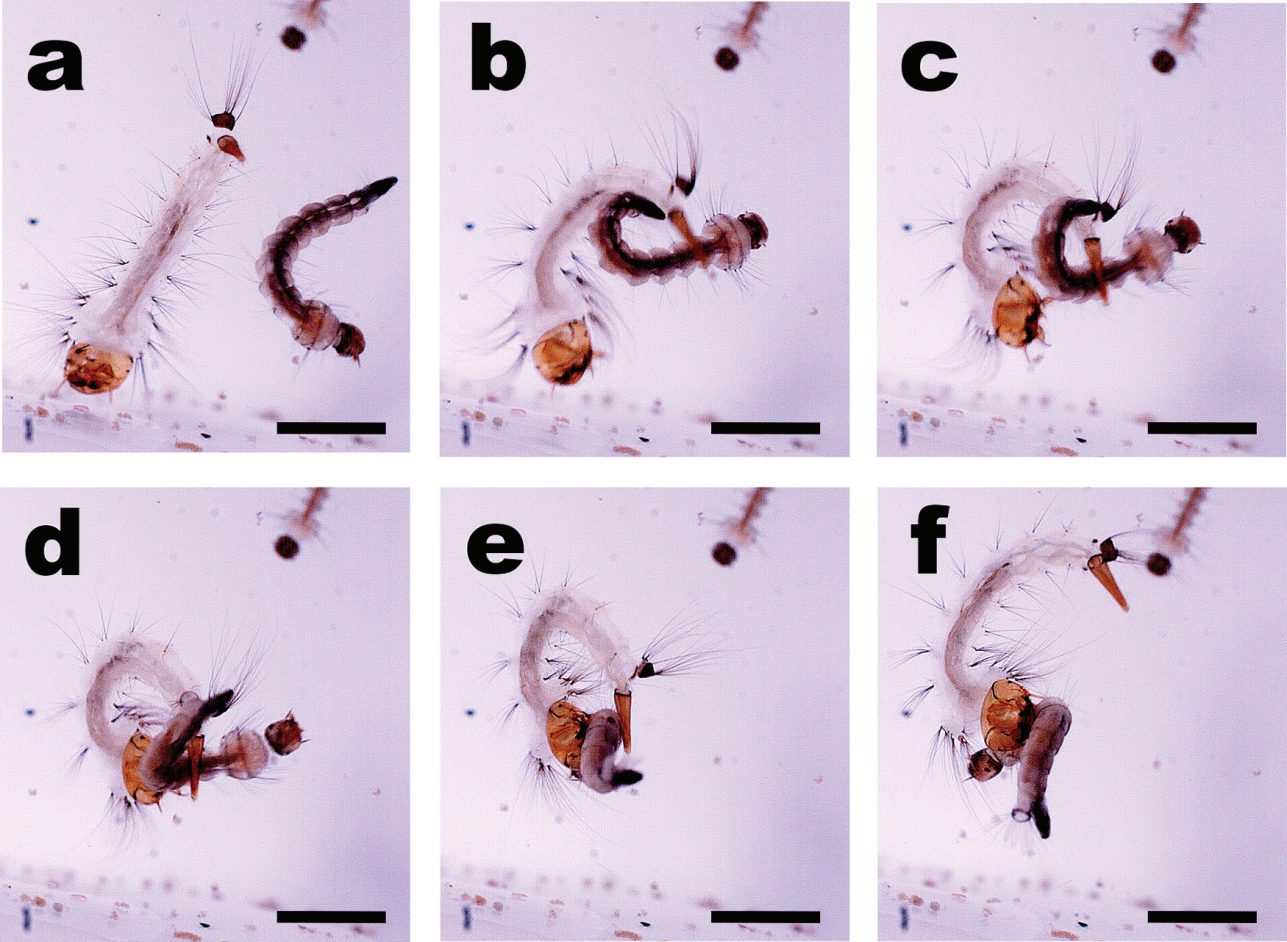
Strike sequence frame grabs of *Sa. cyaneus* on *Ae. aegypti* prey: (a) predator larva (left-light body trunk) with maxillae and mandibles in closed positions and prey larva (right-dark body trunk) in curled position; (b) *Sa. cyaneus* in early siphon prey-snagging arch with maxillae opening; (c & d) *Sa. cyaneus* pulling siphon-snared prey into gaped mandibles and maxillae; (e) *Sa. cyaneus* prey-snagging arch complete and maxillae closed onto prey larvae; (f) *Sa. cyaneus* siphon releases and maxillae clasped on prey and mandibles tearing into prey. Scale = 2 mm.

**Fig. 3.**
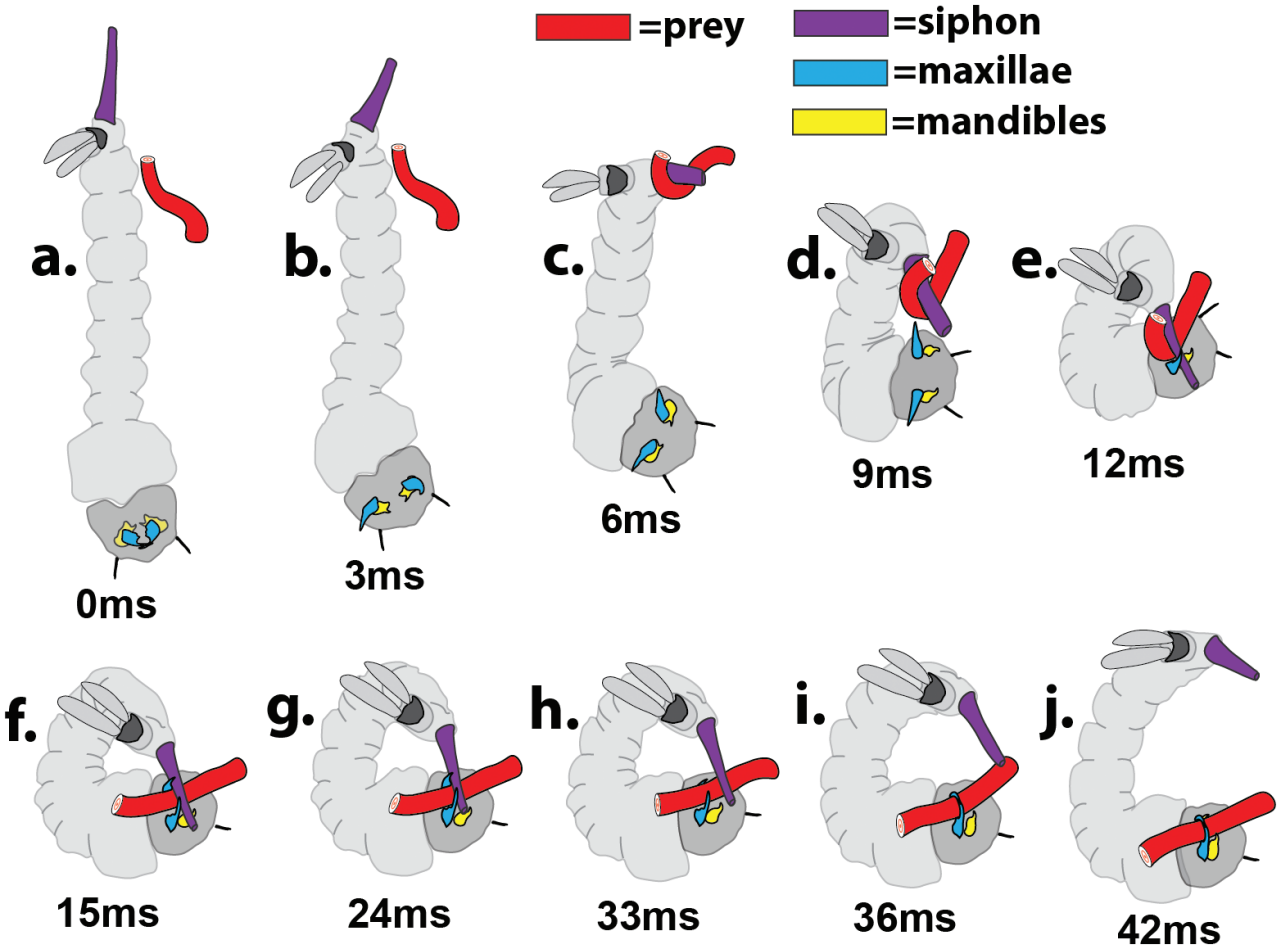
Strike sequence illustration of *Sa. cyaneus* on a flexing larviform prey target. Key structures for *Sa. cyaneus* are the siphon, mandibles and maxillae: (a) positions at rest; (b) slight siphon movement; (c–g) ventrolateral body arch accompanied by siphon hooking the pulling the prey into outstretching maxillae and mandibles; (h–i) maxillae clamped onto the prey which is held parallel to the predator. Running time values from onset of strike movement shown as milliseconds (ms). Strike sequence illustration of *Sa. cyaneus* on a red larviform prey target. Key structures are colored as follows: purple, yellow, and blue for *Sa. cyaneus* siphon, mandibles, and maxillae, respectively. Running time values from onset of strike movement shown as milliseconds (ms).

Larvae tended to be positioned close to the walls or bottoms of containers with their dorsa facing the nearest surface. If on the bottom they actively positioned themselves to lie facing up and if surfaced (respiring) they hang along edges facing in. We saw and/or recorded numerous strikes of prey from both the bottoms and the edges of various containers. The maxillae and mandibles were held at rest against the head capsule in a closed position whereby the apical teeth overlapped (Figs. 1a, 2a, and 3a). Before a strike the maxillae and mandibles were opened and projected out at about 90° angles from the body axis (Figs. 1b, 2b, and 3b).

### *Sa. cyaneus* Strike

The response to a prey larva began with a slight siphon movement to an axial position (Fig. 3b) in line with the body trunk followed by a rapid ventrolateral body arch where the siphon hooked the prey larva and pulled it to the head and into the open and outstretched maxillae and mandibles (Fig. 3c–g). The head and posterior segments rotated so that the ventral surface of the head (and mouthparts) met the siphon with prey without obstruction and interference from the saddle (segment X and papillae). The mechanism brought the siphon flat across the head in a line between the maxillae and mandibles (Fig. 3e–g). The maxillae clamped onto the prey which is then held parallel to the predator (Fig. 3h–j).

### *Sa. cyaneus* Prey Securing and Manipulation

The maxillae had a secure grasp on the prey within 15 ms (Fig. 3f), but release of the siphon and the onset of mandibular movement occurred about 20 ms later (Fig 3h and i). In front of the maxillae, the mandibles are projected out at about 90° (like the maxillae) and repeatedly, but arrhythmically, opened and closed so that their serrated teeth tore into the prey. Aside from what was ingested by the predator, liquified contents of the larval prey could often be seen spilling out of the gaping wound. The degree of prey consumption was highly variable as discarded carcasses ranged from dead, but unconsumed dead larvae to remnants consisting of head capsules and siphons connected by pieces of mangled cuticle.

### *Sa. cyaneus* Prey Species Selection

We conducted a simple experiment to determine if *Sa. cyaneus* can discriminate between prey of other mosquito species and conspecific larvae. Into 28 ice-tray cells (approx. 15 ml water) were placed 1 of each of the following: *Sa. cyaneus* fourth instar larva (6–8 mm), *Sa. cyaneus* second instar larva (2-4 mm), and a second instar *Ae. albopictus* larva (2–4 mm). Within 1 hour, *Ae*. *albopictus* larvae were killed in 27 of the 28 cells of ice-cube trays, but no cannibalism was observed, i.e., all early-stage *Sa. cyaneus* larvae were alive.

### *Sa. cyaneus* Prey Searching

**Fig. 4.**
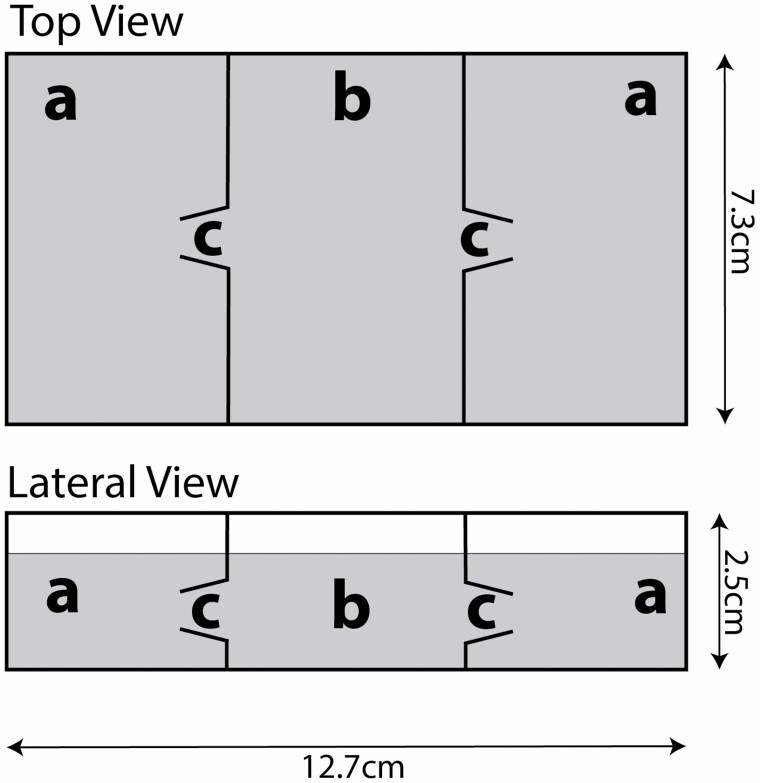
Behavioral bioassay to test for prey searching behavior by fourth instar *Sa. cyaneus* larvae: (a) choice traps receiving either left blank or provisioned with 3–4 *Ae. albopictus* prey larvae; (b) central release chamber for a single *Sa. cyaneus* predator larva; (c) cone gates leading from b to a.

We designed a special choice arena with funnel-gated traps to determine if *Sa. cyaneus* larvae search for prey (Fig. 4). Ten 2nd instar *Ae. albopictus* larvae were placed in one chamber and the opposite one was left blank. Then one 4th instar *Sa. cyaneus* larva was placed in the central release chamber. In 11 trials run in darkness, after 1 h, prey searching was significantly demonstrated as 8 of the *Sa. cyaneus* larvae had entered the *Aedes*-baited trap while 1 went into the blank control and 2 remained in the release chamber (sign test, *z* = 2.33, *p* = 0.02).

### *Sa. cyaneus* Diet and Nutrition

We performed an experiment to compare the nutritional value of diet as measured by total lipid contents of *Sa. cyaneus* larvae on a prey diet versus a ground fish food diet. Like-sized third instar *Sa. cyaneus* larvae were removed from colony pans and placed individually into ice-cube-tray cells. Over a 4-day period, prey-fed predator larvae were maintained on 4–5 *Ae. albopictus* larvae, while filter-feeding larvae were maintained on ground fish food, and unfed control larvae received neither prey nor fish food. Total lipid was then measured of individual *Sa. cyaneus* larvae was then measured by the vanillin phosphoric acid colorimetric method of Van Handel (1985). Mean larval lipid levels in prey-fed and fish-food fed larvae were not significantly different and mean fish-food fed larval lipid was significantly higher than the mean of unfed control larval lipids (Table 1).

**Table 1.** Mean total lipid content of *Sa. cyaneus* larvae kept individually in ice tray cells over 4 d with different food treatments

Food over 4 d	*n*	µg lipid ± SEM^a^
None	22	330.8 ± 15.5a
Ground fish food	29	398.9 ± 14.0b
Prey larvae (*Ae. albopictus*)	27	401.8 ± 32.0ab

Values followed by the same letter are not significantly different, *P* > 0.05 (Tukey–Kramer Method).

### Tx. amboinensis

The structures noticeably involved in prey capture and manipulation in this species included abdominal segments 1 and 2, the neck, the mandibles (Fig. 1c and d), and the LPBs (Fig. 1c–e) that, compared to *Ps. ciliata*, were much more rigid and, therefore, exhibited more consistent movements. (Note: Fig. 1c–e are SEMs of *Tx. brevipalpis* (Theolbald, Dipera: Culicidae) from Linley (1990), used with permission from the Florida Entomological Society. That species is a consubgener of *Tx. amboinensis*) (Supp Videos S6–8 [online only]).

### *Tx. amboinensis* Prestrike Position

*Tx* **.** *amboinensis* larvae maintained variable angles in the water and, unlike *Sa. cyaneus* and *Ps. ciliata* (which maintained a straight body axis), often curved and angled their bodies towards prey. As in *Ps. ciliata*, at rest their mandibles remained closed and overlapping and the LPBs closed and folded ventrally against the head capsule and inside of the antennae which were directed forward.

### *Tx. amboinensis* Strike

**Fig. 5.**
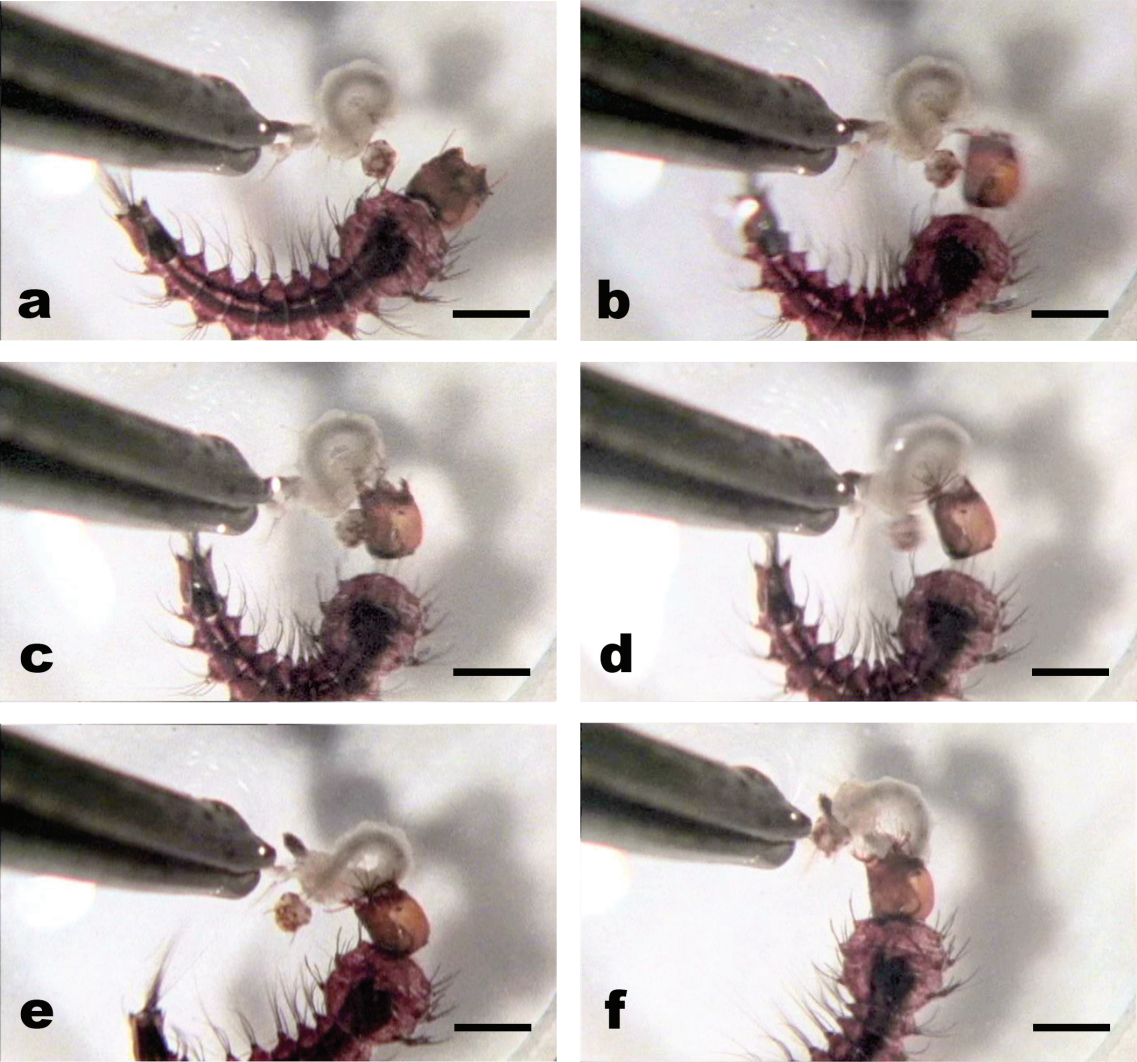
Strike sequence frame grabs of *Tx. amboinensis* on tethered *Ae. aegypti* prey: (a) LPBs at resting position with antennae forward and neck not extended; (b) strike begins as neck extends (notice constriction of anterior abdominal segments) and LPBs swing out to open position; (c) neck further extended with LPBs fanning and gaping mandibles clearly visible; (d) LPB basket fully formed and grasping prey as mandibles tears into prey; (e) LPB basket and mandibles tightly grasp prey as the head is returned to normal position against the thorax; (f) LPB basket retracting and mandibles occluding. Scale = 2 mm.

Unlike *Sa. cyaneus*, but similar to *Ps. ciliata*, *Tx. amboinensis* struck only from surface positions (with their siphons penetrating the surface film) (Linley 1990). Unlike *Ps. ciliata*, which typically struck in a straight-ahead (axial-linear) fashion, *Tx. amboinensis* strikes often involved a great deal of both body arching and head twisting (Linley 1990, provides a detailed account of these variations) (see Fig. 5 and Suppl Materials [online only]). Independent of strike angles and head and body position, all strikes began as the anterior abdominal segments became constricted (Figs. 5b–e and 7b–e) and the neck extended and the head capsule was thrust towards the prey in a mechanism that appeared to involve hemostatic pressure. Reflecting variations in the degree of body arching during strikes, abdominal constrictions exhibited variation, with the consistent feature being that head extension co-occurred with the axial contraction of at least 1 of abdominal segments 1–3. Figure 7 illustrates a “frontal with head extension” strike (Linley 1990) that represents a composite of observed head-propulsion-coupled abdominal contractions: At full extension (Figs. 5d and 7e) the width from a dorsal view (Fig. 7*y2*) shows a range of decrease in widths from the pre-extension widths (Fig. 7*y*1). At this time the antennae, LPBs, and mandibles dramatically abducted into the strike position: the antennae angled out nearly perpendicular from the head capsule to accommodate the LPBs, which first flipped up and out to an “open position” just above the frontal plane and approx. 120° out and forward from the head (Figs. 5b and 7e). Then the LPBs dramatically fanned out to form 180° baskets composed of the 9 rigid and toothed elements (composed of fused setae) from each side of the head (Figs. 5d–f, 7f, and 8a). As the LPBs opened, the mandibles opened wide (with the distance between the innermost teeth being as wide as the head capsule). The impact of strikes, although apparently considerably less forceful than in *Ps. ciliata*, was marked by the simultaneous clamping/closing movements of the mandibles and variable points of contact of elements of the LPBs with the body of the prey.

### *Tx. amboinensis* LPB Retraction

After the prey was firmly grasped by the mandibles, the LPBs returned to their resting positions in a reverse of the 2-step (opening) fanning process previously described, and the antennae moved back to their typical forward-pointing positions (Fig. 5f). The antennae then moved forward to their pre-strike positions.

### *Tx. amboinensis* Prey Securing and Manipulation

The mandibles had a secure grasp on the prey within 15 ms (Figs. 5e and 7f). As in *Ps. ciliata*, the mandibles appeared to be the only structures that manipulated the prey as it was consumed.

### Ps. ciliate

The structures noticeably associated with the capturing and manipulation of prey included (from posterior to anterior) abdominal segments 1 and 2, the neck, the robust and sharply-toothed mandibles, and the lateral palatal brushes (LPBs), which are composed of independent modified setae (Fig. 1f). The antennae moved stereotypically as well, but without contacting the prey (Supp Videos S9-10 [online only]).

### *Ps. ciliata* Prestrike Position

*Ps. ciliata* larvae maintained a low angle with the surface of the water. Their head structures were maintained at rest with the mandibles closed and overlapping, the antennae pointing outward perpendicular to the body axis, and the LPBs closed and folded backwards and down against the head capsule. The abdomen appeared full and relaxed, with segments 1 and 2 exhibiting similar spacing and compression to the rest of the abdominal segments. As larvae of their sister species *Ps. howardii* actively search for prey (Lounibos 2001), *Ps. ciliata* tended to align themselves (both horizontally and vertically) in a direct line towards prey larvae.

### *Ps. ciliata* Strike

**Fig. 6.**
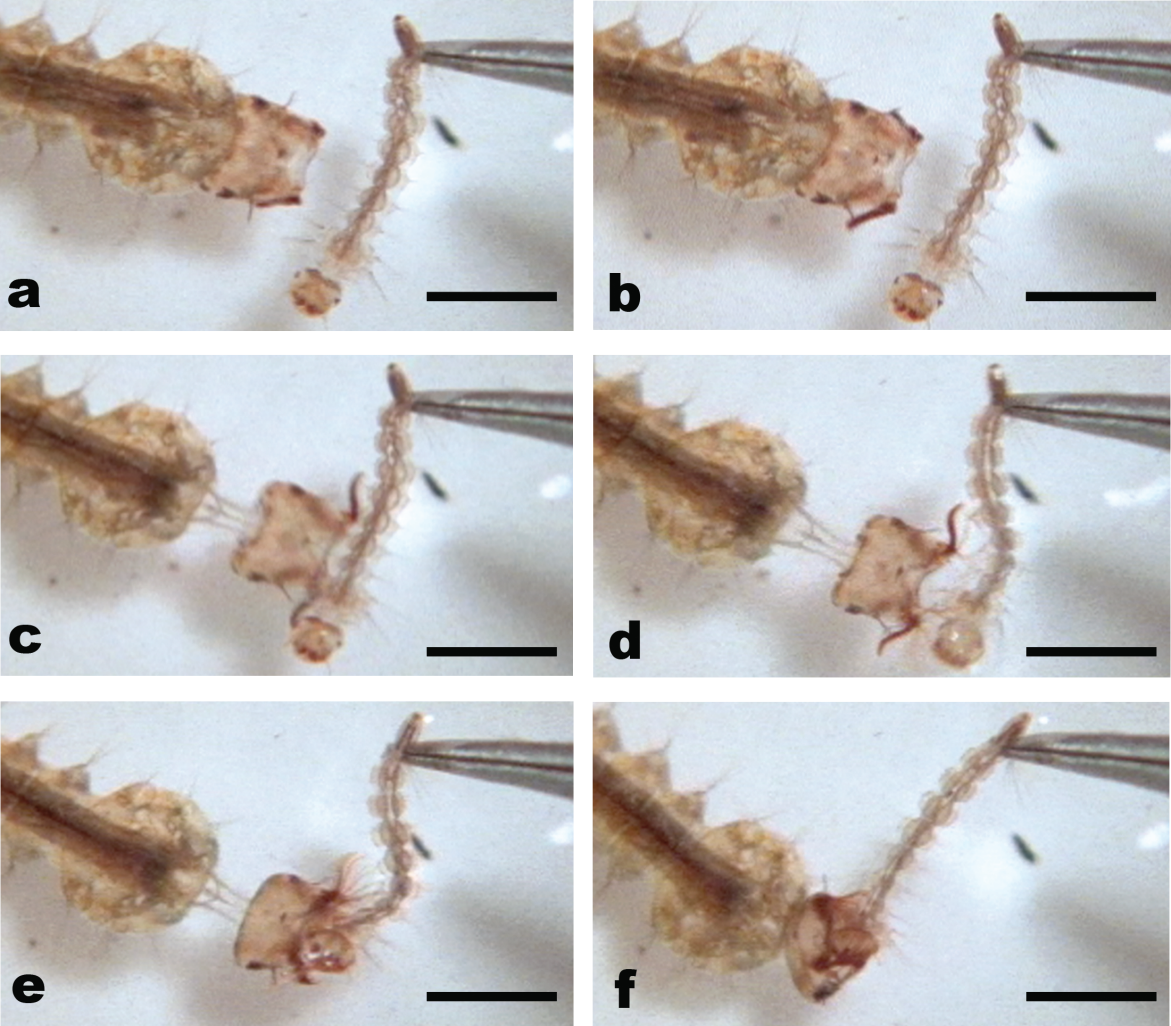
Strike sequence frame grabs of *Ps. ciliata* on tethered *Ae. aegypti* prey: (a) LPBs closed in resting position and head in normal position against the thorax; (b) LPBs opening; (c) neck extension/head propulsion (note the contraction of abdominal segment 1 in the upper left of the frame) and LPBs in “open position” (pre fanning); (d) peak of neck extension, mandibles gaping and some LPB fanning; (e) gaping mandible closing and extensive LPB fanning to form basket; (f) head retraction, mandibles closed, and LPB elements folded down and forward from their fanned position more tightly onto the prey. Scale = 2 mm.

**Fig. 7.**
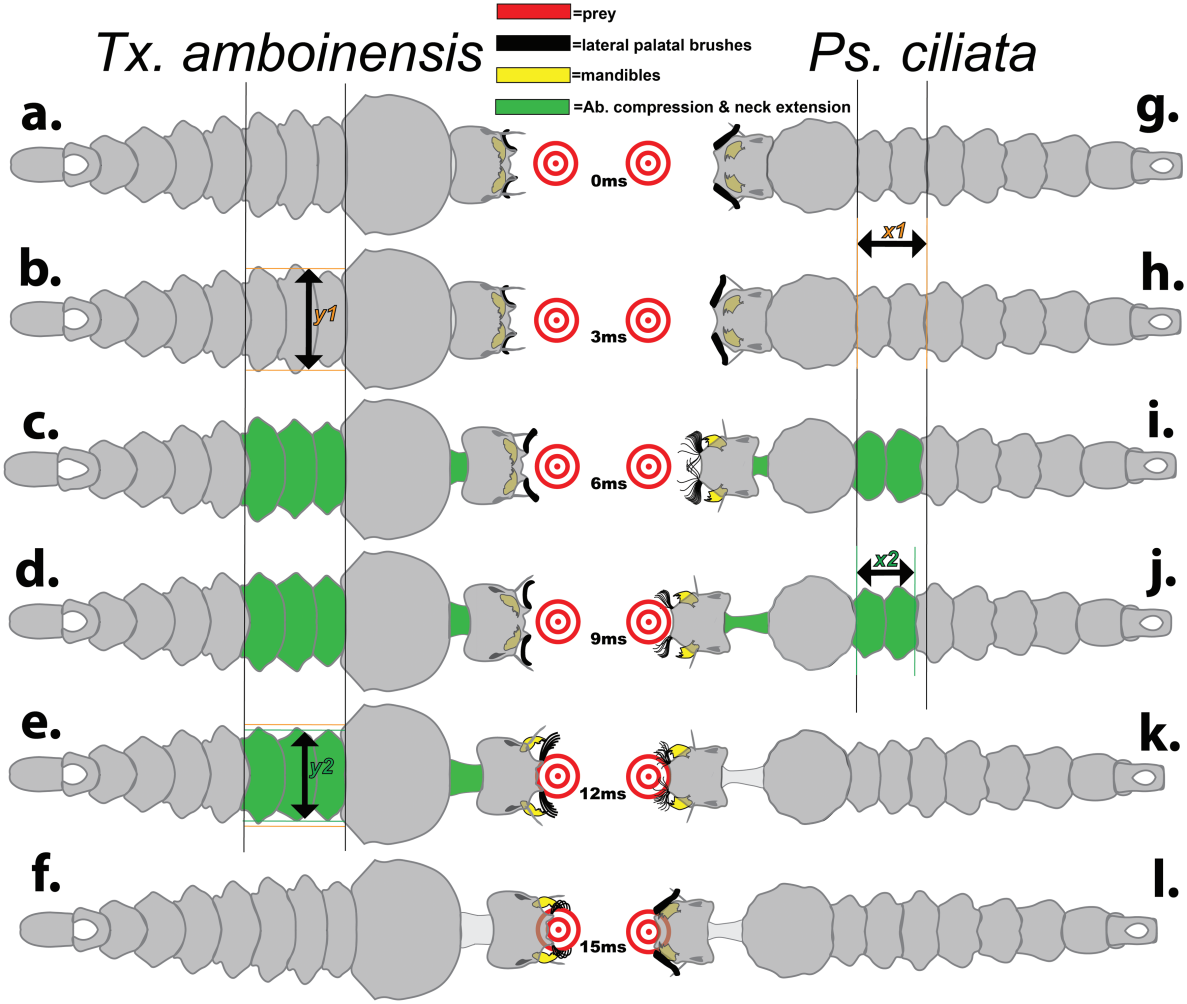
Strike sequence on a bullseye prey target for *Tx. amboinensis* (a–f on left side) and *Ps. ciliata* (g–l on the right side). Key structures are the mandibles, LPBs, and necks. *y1* and *y2* represent changes in length due to radial compression in *Tx. amboinensis* abdominal segments I–III while *x1* and *x2* represent changes in length due to axial compression in *Ps. ciliata* abdominal segments I and II. Running time values from onset of strike movement shown as milliseconds (ms).

**Fig. 8.**
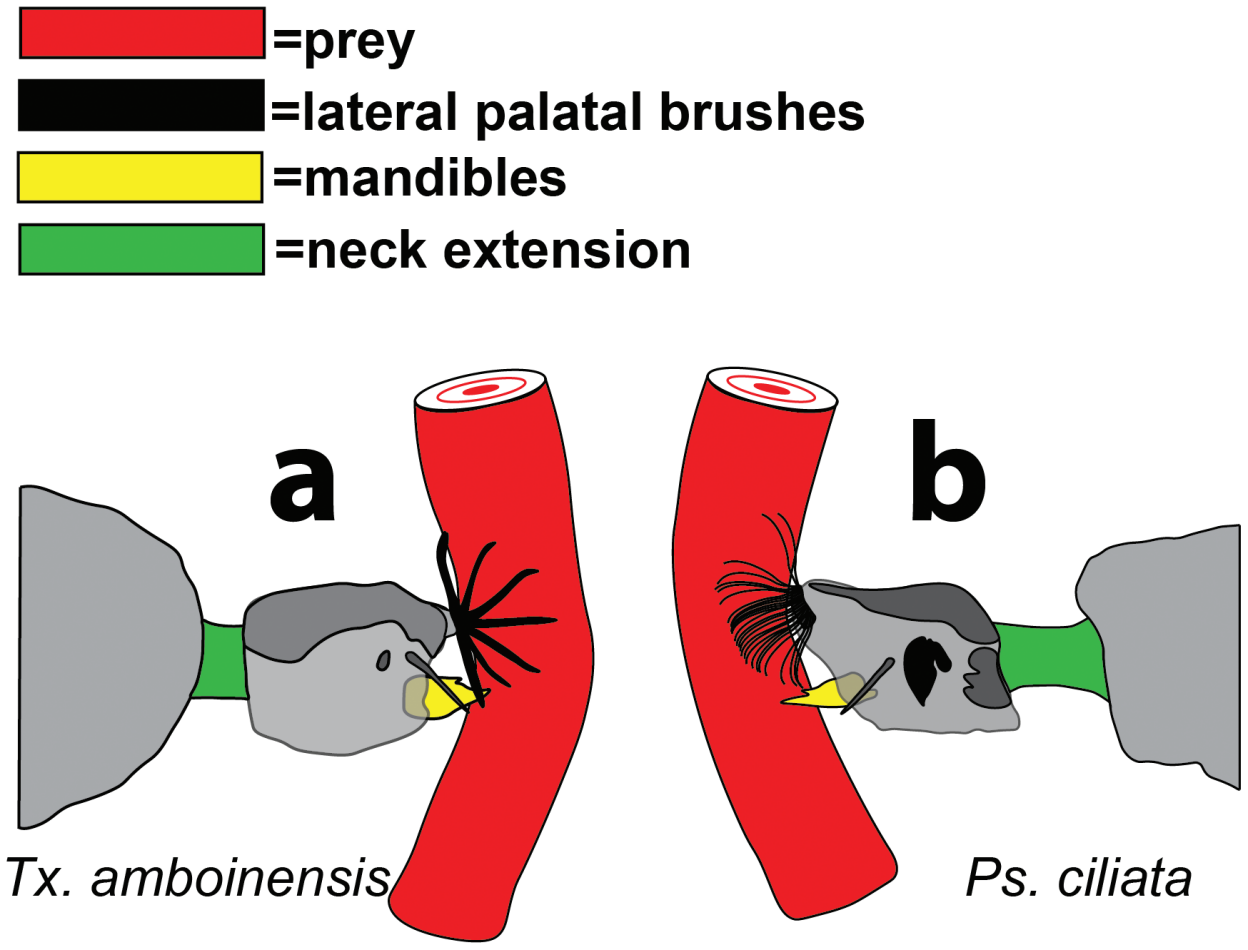
Lateral view when prey (flexing larviform target) is secured showing splayed LPBs, mandibles and extended necks: (a) *Tx. amboinensis*; and (b) *Ps. ciliata*.

Unlike *Sa. cyaneus*, *Ps. ciliata* did not strike from submerged positions (siphons not penetrating the surface film). An accordion-like compression of abdominal segments 1 and 2 (Figs. 6c and d and 7h–j) was accompanied by a dramatic propulsion of the head capsule towards the prey by a presumably hemostatic mechanism (Figs. 6c–e and 7h–j). At full extension (Figs. 6d and 7j) the dorsal length (Fig. 7*x2*) decreased approximately 20% from its pre-extension length (Fig. 7*x*1). Thus, abdominal segments 1 and 2 were compressed to approximately 80% of their length, and the neck extension reached to approximate the length of the head capsule. As the head shot forward, the mandibles and the LPBs simultaneously moved to an “open position” approximately 35° forward from their prestrike positions, and the antennae moved forward, closing the angle with the body axis to nearly 45° (Figs. 6c and d and 7i). Then the LPBs broadly fanned out to form a flimsy basket-like arrangement around the dorsal half of the front end of the head capsule. This fanning was incomplete as some elements remained together (Figs. 6d and e, 7i, and 8b). The blunt and forceful impact displaced the prey as the mandibles clamped down onto and cut into it, and various setae of the LPBs folded onto the prey. Ultimately, the LPB elements folded down and forward from their fanned position more tightly onto the prey (Figs. 6f, 7k, and 8b).

### *Ps. ciliata* LPB Retraction

The LPBs returned to the prestrike positions back against the head capsule in a graceful 3-step movement. First the elements closed together tightly, but in the open position. Second, the LPBs simultaneously moved forward down and partially fanned-out, contacting the prey, which is held by the mandibles. Finally, the LPBs simultaneously lifted, closed, and folded back into their original positions against the head capsule.

### *Ps. ciliata* Prey Securing and Manipulation

The mandibles had a secure grasp on the prey within 15 ms (Fig. 7l). As in *Tx. amboinensis*, once in the grasp of the mandibles (during and after LPB retraction), prey manipulation into the mouth was performed by the mandibles (and possibly maxillae) until the prey was entirely consumed.

## Discussion

### Convergent Evolution of Prey Capture and Re-evaluation of Mechanisms

Despite their distant relatedness in different tribes of the Culicidae and dissimilar life histories, the obligate predators *Tx. amboinensis* and *Ps. ciliata* have apparently converged on a similar mechanical strategy for preying on mosquito larvae (Fig. 7). By utilizing high speed macrocinematography with sufficient light and magnification to resolve details in the movements of head structures and anterior-abdominal segments, we clearly elucidated dynamic head-propulsion mechanisms that appear to be dependent on hemostatic pressure generated in the anterior abdominal segments. Furthermore, prey grasping at the end of head-propulsion involves simultaneous cooperation of both the mandibles and the LPBs, although in *Ps. ciliata*, the relative flimsiness of the individual brush rays suggests that they may play a lesser role than in *Tx. amboinensis*. In the case of species of Toxorhynchites, this resolves an old debate: Breland (1949) using only a microscope reported that the mandibles of *Tx. septentrionalis* (Dyar & Knab, Diptetra: Culicidae) (=*Tx. rutilus*) alone were used while Linley (1990) using high speed macrocinematography with lower magnification (than used in this study) and a lower-light/silhouetted image reported that the prey of *Tx. amboonensis* and *Tx. brevipalpis* is first captured with the lateral palatal brushes and then transferred to the mandibles. Clearly Linley (1990) wins the debate as his description correctly identifies the involvement of LPBs, but depending on the position of the prey, numerous sharp points including the distal teeth of the brushes and/or the teeth of the mandibles will strike the prey larva and therefore “transfer” to the latter is not indicated.

The consistent and extremely short-lived formation of rigid prey-capturing baskets composed of the LPBs of *Toxorhynchites* is a most impressive and unusual phenomenon that has escaped the notice of previous observors. Breland (1949) never saw the LPBs even move from their at-rest positions while Linley (1990), with slow-motion equipment, noticed the LPBs flip out to the 90° position (what he referred to as “opened”), but never saw the fanned-out baskets. The duration of this basket-position may be related to the size and/or fight of the prey. That our prey larvae were held by forceps could have resulted in longer basket durations. Regardless, this unusual adaptation for prey capture warrants detailed functional-anatomical study. However, freezing these structures in the open position using traditional methods may prove to be impossible.

### Hemostatic Pressure in Prey Capture and Other Uses in Soft-bodied Insects

Hemostatic head-propulsion as a predatory mechanism has not been previously described, although Linley (1990) attributed “internal pressure” or “hydrostatic pressure” as the cause of head extension by *Tx. brevipalpis* and *Tx. amboinensis* during frontal strikes by these predatory species. Dramatic forceful expulsions due to generated hemostatic pressure on internal compartments are found in other soft-bodied insect larvae and adults: The hesperiid caterpillar *Calpodes* (*Hesperia*) *ethlius* (Stoll, Lepidoptera: Hesperidae) expels frass at an impressive mean velocity of 1.3 m/s^−1^ (Caveney et al. 1998). “Nasute” castes of termites squirt noxious allomones at potential predators (Nutting et al. 1974). Clearly the fundamental features of a resilient yet flexible exocuticle, hemolymph, and dynamic longitudinal and circular musculature provide the basis for generating tremendous internal pressure. In the case of *Ps. ciliata* and *Tx. amboinensis*, the projectile is the head and, being continuous with the hemocoel via the neck, is essentially “tethered” to the body.

### Prey Capture in *Sa. cyaneus* and Related Sabethine Facultative Predators

*Sa. cyaneus* on the other hand, is a facultative predator that is clearly much less adapted for the capture and consumption of prey, but their use of the siphon and the full sweeping arch of the larval body enables a predator to prey range that is certainly larger than the ranges of *Tx. amboinensis* and *Ps. ciliata*. On the other hand, with head propulsion mechanisms, *Tx. ambionensis* and *Ps. ciliata* may be capable of closer range capture. Van den Assem (1959) described similar maxilla-clasping functions in the cannibalistic behavior of two species of old-world sabethine mosquitoes of the genus *Tripteroides*. After prey capture, extraction of nutritious tissues and fluids by *Sa. cyaneus* appears awkward and inefficient. That many dead prey larvae appear largely intact suggests that once dropped, a carcass will not be recovered. It is possible that *Sa. cyaneus* are murdering their competition or “surplus killing,” as occurs in larvae of *Toxorhynchites* spp. (Corbet 1985) and of the predatory frog-biting midge *Corethrella appendiculata* (Grabham, Diptera: Corethrellidae) (Lounibos et al. 2008). Furthermore, dead prey larvae may serve to spike the micronutrient cycle in the container habitat: In controlled experiments *Drosophila melanogaster* (Meigen, Diptera: Drospohilidae) carcasses in larval containers increased survivorship, development time, and mass in *Ae. aegypti* and *Ae. albopictus* (Daugherty et al. 2000).

In the tribe Sabethini maxillary modifications for grasping are conspicuous and suggest that facultative predation is quite common and morphologically diverse. Three modifications of maxillae have been described: bundles of spicules, elongate apical teeth, and claws (Harbach and Peyton 1993). Elongate apical teeth on the maxillae are characteristic in 9 of the 14 genera: *Kimia* (Harbach et al. 1997), *Tripteroides* (Van den Assem 1959), *Runchomyia* (Zavortink 1979b), some *Wyeomyia* (Motta & Lourenco-de-Oliveira 2000), *Isotomyia* (Harbach & Peyton 1993)*, Shannoniana* (Talaga et al 2016, Harbach & Peyton 1993), *Johnbelkinia* (Zavortink 1979b), and all 5 of the subgenera of *Sabethes* (Harbach & Peyton 1993). Further, maxillary apical claws, in addition to long apical teeth, are found in *Isotomyia*, *Shannoniana*, and the *Tripteroides* subgenus *Rachisoura*, and maxillary bundles are found in *Topomyia*, *Johnbelkinia*, and *Runchomyia* (Harbach & Peyton 1993). *Trichoprosopon* lack maxillary adaptations for grasping, but feature elongate mandibles (Zavortink 1979a).

Although previous workers have identified predation by direct observation or circumstantial evidence, our high-speed analysis of the mechanism of prey capture by *Sa. cyaneus* is the first to show how maxillary grasping modifications are actually used. Further, the use by *Sa.* (*Sab.*) *cyaneus* of the siphon to snag prey is the first report of larval structures other than mouthparts or antennae being used by predatory Culicimorpha to capture and manipulate prey. Ongoing work in our laboratory indicates that *Sa. (Sbo.) chloropterus* (von Humboldt, Diptera: Culicidae) capture prey with an identical siphon-snagging mechanism (Boyd & MacFadden, personal communication). Of course, much high-speed motion analysis work needs to be done on other maxilla-modified sabethines to determine just how widespread that siphon-snagging is in this tribe.

### Larval Predation Mechanisms as Components of Complex Life Histories

The role of the adult mosquito in selecting sites to deposit eggs should not be discounted as it marks the beginning predatory larval stage of a complex life history. As noted formerly, *Psorophora* (*Psorophora*) are exemplars of a boom-or-bust strategy in short-lived flood pools, in which larvae die if starved, and adults are relatively short-lived (Lounibos 2001). By contrast, larvae of *Toxorhynchites* and *Sabethes* that occur in more stable habitats, have considerably longer larval lifespans than *Psorophora* (*Psorophora*), may resist starvation in some larval stages, and are relatively long-lived as adults (Lounibos 2001, Galindo 1958, Galindo et al. 1958). The larval-starvation resistance of fourth instar *Toxorhynchites* spp. facilitates a delay in metamorphosis until a critical weight for pupation is achieved (Lounibos 1979), and that feature contributes to the longevity of adults, whose females are unable to consume blood (Steffan and Evenhuis 1981).

One limitation of the current study is the absence of observations of predation on frequently available alternative prey, which accounted for the majority of arthropods consumed in the only study of prey of *Tx. rutilus* in nature (Campos and Lounibos 2000a). Wilkerson et al. (2021) state that mosquito larvae account for the majority of prey eaten by *Ps. ciliata* in nature, but as the origins of this claim are unknown, research on the gut contents of wild-caught *Ps. ciliata* larvae is needed to describe the larval diet of this species in nature, ideally in multiple locations and times.

The spectrum of sabethine mouthpart adaptations for prey capture is consistent with the hypothesis of Talaga et al. (2016) that facultative predation in the tribe Sabethini evolved to accommodate growth and development of their larvae in small but abundant phytotelm aquatic habitats, where larval food resources are often limited (Kitching 2000). Although *Sa. cyaneus* immatures are most commonly found in water-containing treeholes and bamboo internodes (Galindo et al. 1958), which are among the largest (by volume) of phytotelm habitats (Talaga et al. 2016), the trade-off between resource limitation and a secure larval habitat, is consistent with their hypothesis about the frequency of intraguild predation in all Sabethini, which are common occupants of phytotelmata, especially in the Neotropics. Our experiments also confirmed Galindo et al. (1958) that cannibalism is relatively rare in *Sa. cyaneus*, and that facultative predation may be primarily a failsafe strategy in this species when alternative larval resources are scarce. Although physical examination of prey remains in midguts of wild *Sa. cyaneus* would not be practical because prey items are not usually consumed whole by this species (Fig. 3), DNA-based probes of gut contents of wild-caught larvae could yield relevant information on the frequency and importance of predation by this species in nature.

## Future Directions

Campos and Lounibos (2000a) identified the exoskeletal remains of terrestrial insects from nine orders, plus terrestrial mites and spiders, in the dissected midguts of *Tx. rutilus* larvae collected from natural treeholes and discarded tires containing water. It would be informative to examine how larvae of this and other obligatory predatory species adapt their underwater prey-capture mechanisms, described in this study for mosquito prey, to terrestrial arthropods that land upon and are snared from the water surface. Our results on mechanisms of facultative predation by *Sa. cyaneus*, also set the stage for more detailed studies of predation by other sabethine species known to have modified mouthparts and a demonstrated capacity to predate facultatively in the laboratory, e.g., *Runchiomyia magna* (Theobald, Diptera: Culicidae), *Sabethes undosus* (Coquillett, Diptera: Culicidae), *Shannoniana fluviatilis* (Theobald, Diptera: Culicidae), and *Trichoprosopon pallidiventer* (Lutz, Diptera: Culicidae) (Talaga et al. 2016). An unanswered question resolvable with the high-speed cinematography used in our study is this: Do all these species use the siphon to snag motile prey and guide them to their mouthparts? Last but not least, in the context of convergent evolution towards an optimal capture strategy by obligate predators, do species of *Lutzia* use the same three-stage approach identified in this paper for *Tx. rutilus* and *Ps. ciliata*?

## Supplementary Material

saac017_suppl_Supplementary_Video_S1

saac017_suppl_Supplementary_Video_S2

saac017_suppl_Supplementary_Video_S3

saac017_suppl_Supplementary_Video_S4

saac017_suppl_Supplementary_Video_S5

saac017_suppl_Supplementary_Video_S6

saac017_suppl_Supplementary_Video_S7

saac017_suppl_Supplementary_Video_S8

saac017_suppl_Supplementary_Video_S9

saac017_suppl_Supplementary_Video_S10
